# Supplementary material for: Development of quantitative and continuous measure for severity degree of Alzheimer’s disease evaluated from MRI images of 761 human brains
Source: BMC Bioinformatics. 2022 Aug 29;23:357. doi: 10.1186/s12859-022-04903-8 (PMC9422149; doi:10.1186/s12859-022-04903-8)
Supplement: Supplementary file 1 — Additional file 1: Supplementary Figures and Tables. [file 12859_2022_4903_MOESM1_ESM.docx]

**Additional file 1**

**Development of quantitative and continuous measure for severity degree of Alzheimer’s disease evaluated from MRI images of 761 human brains**

Sangyeol Kim, Seongjun Park and Iksoo Chang and the Alzheimer’s Disease Neuroimaging Initiative

**Additional Figures**

**
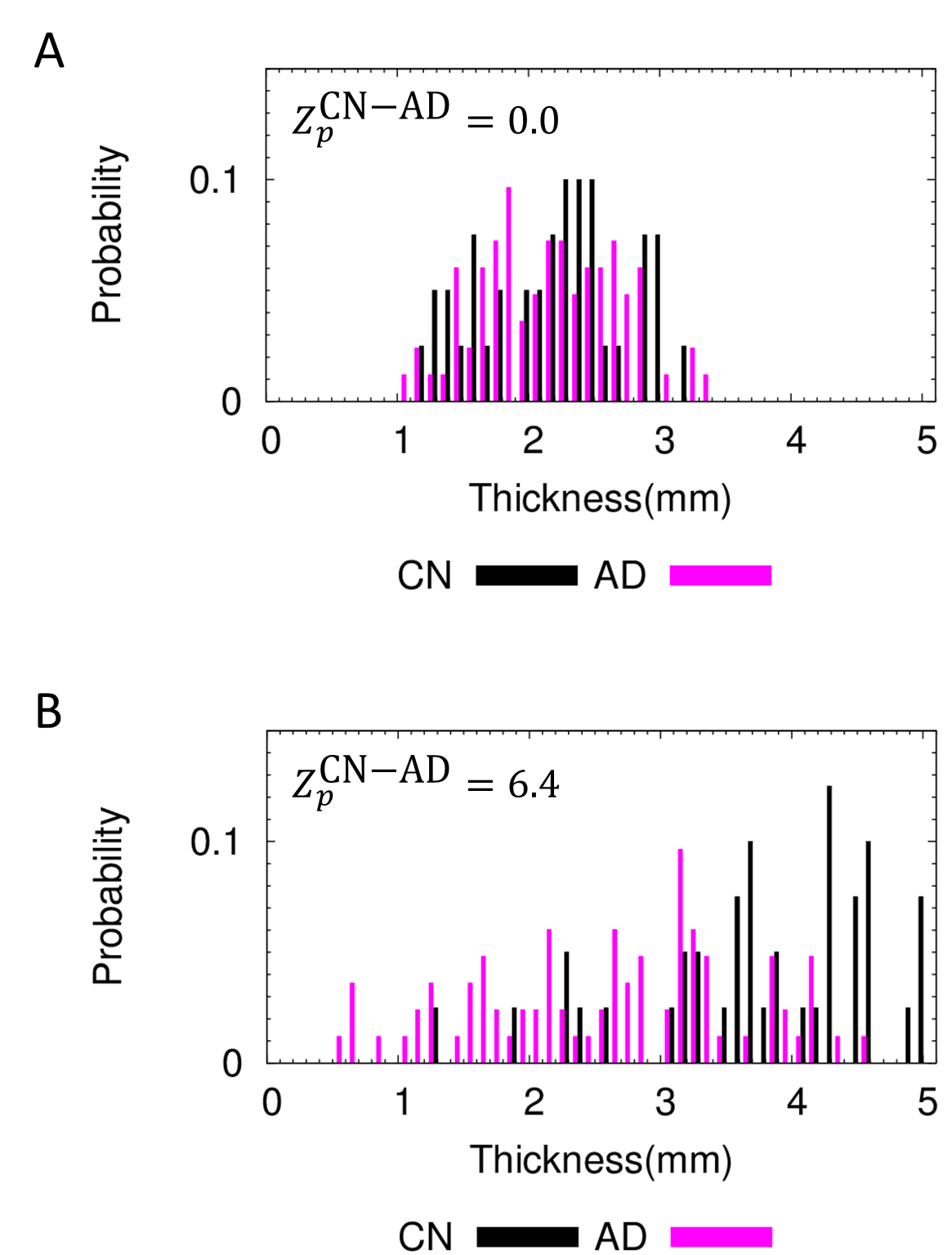
**

**Fig S1. The distribution of cortical thickness of CN and AD subjects in group D of average cortical thickness.** (A) At the smallest $\left| Z_{p}^{\text{CN-AD}} \right|$ value, the two distribution curves of CN and AD subjects are not distinguished. On the other hand, (B) at the largest $\left| Z_{p}^{\text{CN-AD}} \right|$ value, the two distribution curves are distinguished.

**
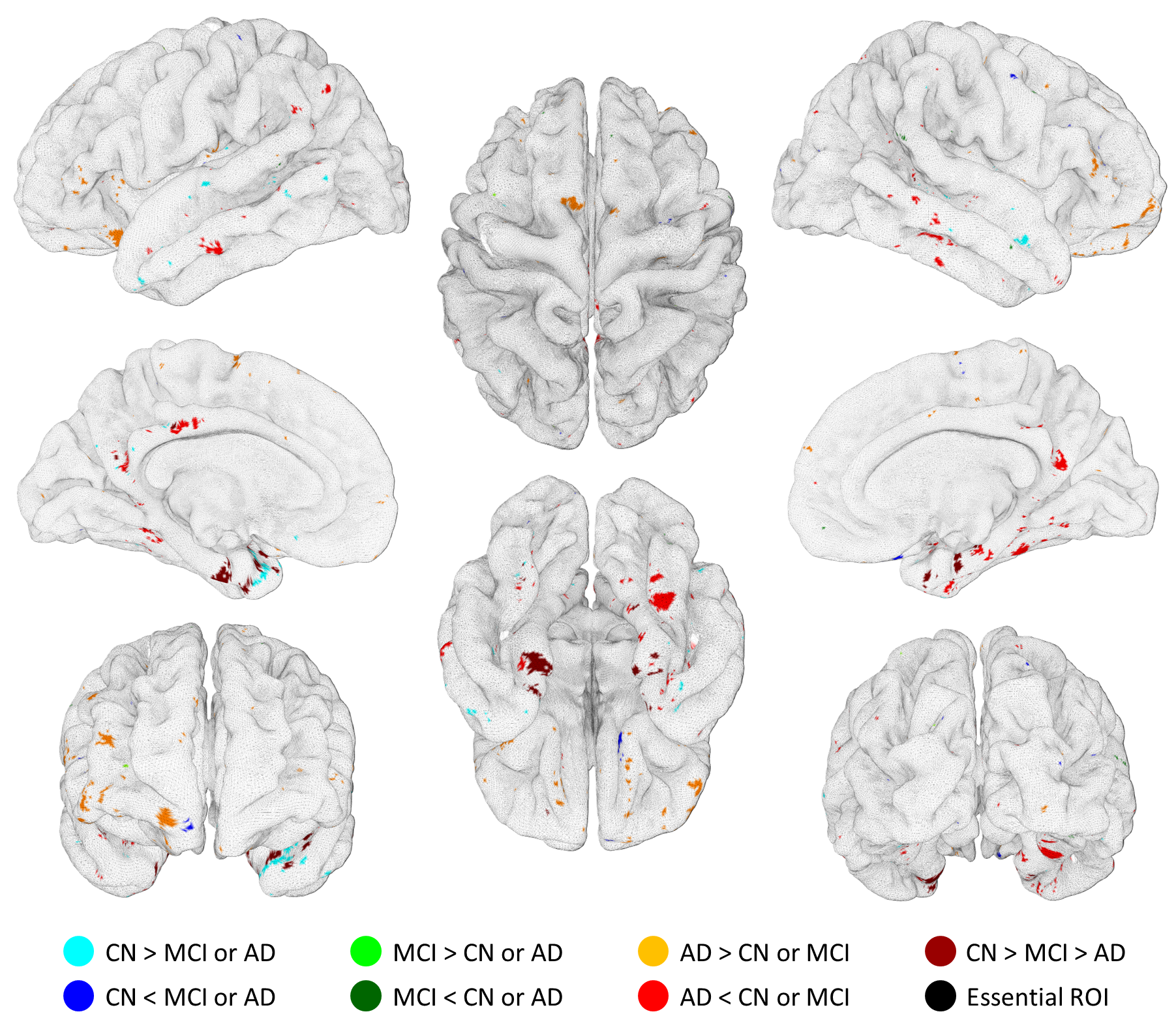
**

**Fig S2. ROI vertices found commonly more than three times from the four groups of the average cortical thickness.** ROI vertices at which the cortical thickness of CN subjects are thicker (thinner) are represented by cyan (blue) color. ROI vertices at which the cortical thickness of subjects with MCI are thicker (thinner) are represented by green (dark green) color. ROI vertices at which the cortical thickness of subjects with AD are thicker (thinner) are represented by orange (red) color. The ROI vertices at which the cortical thickness decreases in the descending order of CN-MCI-AD are represented by dark red.

**
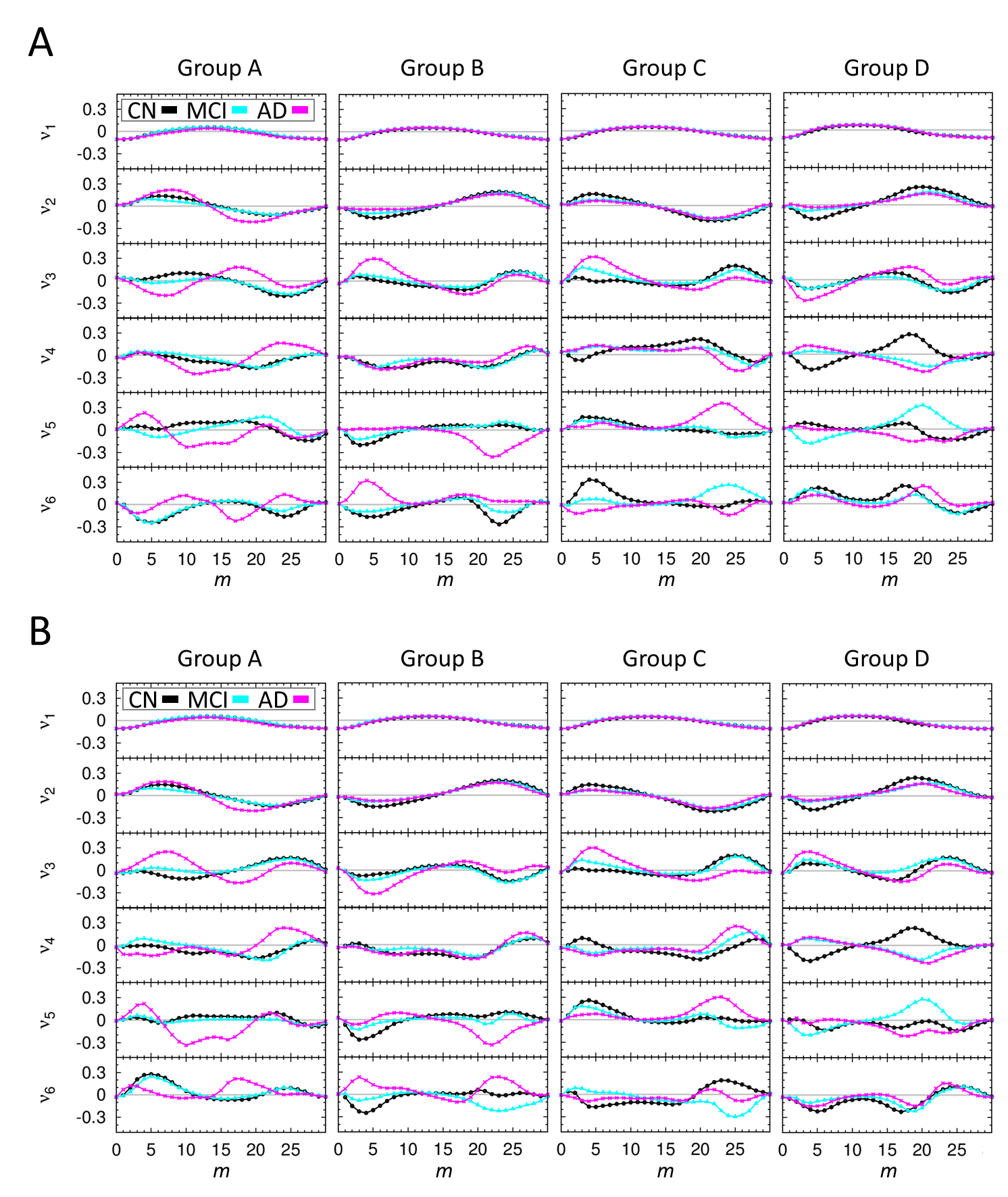
**

**Fig S3. The character of score matrix for each group of average cortical thickness.** (A) The results of singular value decomposition analysis on score matrices, which are composed of 365 CN, 481 MCI, 165 AD human brain images as a training set and used for the second iteration of stratified 3-fold cross validation test. And (C) the results of that which are composed of 366 CN, 483 MCI, 166 AD human brain images as a training set and used for the third iteration of stratified 3-fold cross validation test. For each group of average cortical thickness, six singular vectors corresponding to the six largest singular values are presented. Here, x-axis is *m* value defined in the third section of methods, and y-axis is an arbitrary unit for the singular vectors. For each graph, the singular vector components for CN, MCI, and AD subjects are plotted by black, cyan, magenta colors, respectively.

**
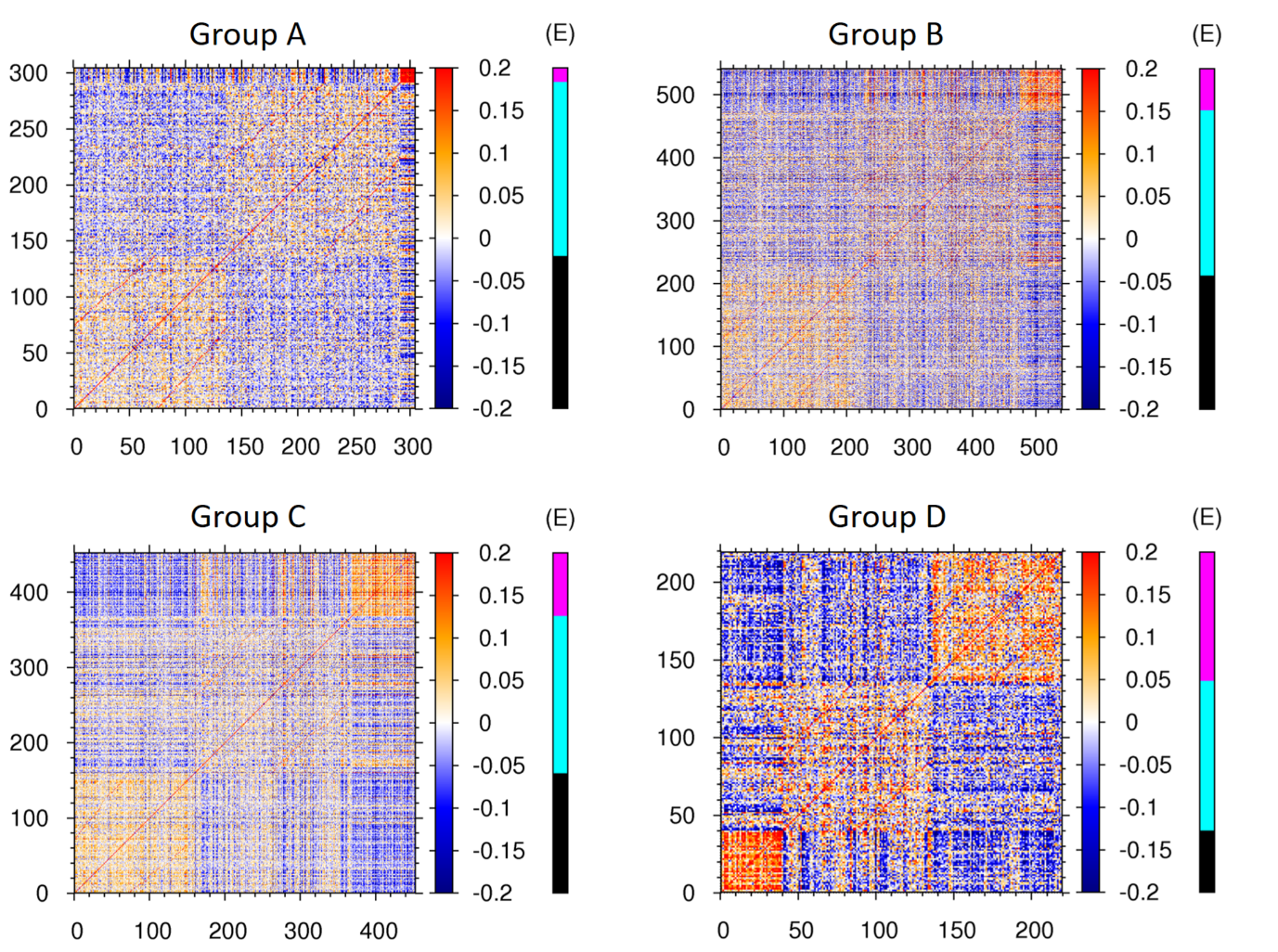
**

**Fig S4. Covariance correlation matrices for each group of cortical thickness.** The heat maps is the covariance correlation matrix for group A to D of average cortical thickness. The extra E-cohort color bar at the right of the heat maps represent the clinically determined cohort of cognitively normal subjects and subjects with mild cognitive impairment and Alzheimer’s disease denoted by black, cyan, and magenta colors, respectively.

**Additional Table**

|  | | | | | |
| --- | --- | --- | --- | --- | --- |
| **Table S1. Upper / lower cut-off *Z* score and number of essential ROI vertices** | | | | | |
|  | Z^CN-MCI^ | Z^CN-AD^ | Z^MCI-AD^ | Number of  essential ROI vertices |  |
| Group A | 4.4 / -4.1 | 6.3 / -5.0 | 6.4 / -5.1 | 479 |  |
| Group B | 4.7 / -3.9 | 6.1 / -4.4 | 4.8 / -4.3 | 520 |  |
| Group C | 4.9 / -4.4 | 8.7 / -4.3 | 5.7 / -4.2 | 494 |  |
| Group D | 4.2 / -4.2 | 5.5 / -4.6 | 4.7 / -4.2 | 564 |  |
| AD, Alzheimer’s disease; CN, cognitively normal; MCI, mild cognitive impairment; ROI, region-of-interest. | | | | |  |

**Table S2. Confusion matrix for the results**

| Self-recognition test | | | | | | | | | | | | | | | | | | | | | | | | | | |
| --- | --- | --- | --- | --- | --- | --- | --- | --- | --- | --- | --- | --- | --- | --- | --- | --- | --- | --- | --- | --- | --- | --- | --- | --- | --- | --- |
|  | | True Positive | | | False Positive | | | False Negative | | | True Negative | | | Accuracy | | | Recall | | | Precision | | | F1-score | | |  |
| CN | | 507 | | | 168 | | | 40 | | | 801 | | | 86% | | | 93% | | | 75% | | | 83% | | |  |
| MCI | | 502 | | | 37 | | | 220 | | | 757 | | | 83% | | | 70% | | | 93% | | | 80% | | |  |
| AD | | 227 | | | 75 | | | 20 | | | 1194 | | | 94% | | | 92% | | | 75% | | | 83% | | |  |
|  | | |  | | |  | | |  | | |  | | |  | | |  | | |  | | |  | | |
| Stratified 3-fold cross validation test | | | | | | | | | | | | | | | | | | | | | | | | | | |
|  | True Positive | | | False Positive | | | False Negative | | | True Negative | | | Accuracy | | | Recall | | | Precision | | | F1-score | | |  |  |
| CN | 463 | | | 222 | | | 84 | | | 747 | | | 80% | | | 85% | | | 68% | | | 75% | | |  |  |
| MCI | 430 | | | 96 | | | 292 | | | 698 | | | 74% | | | 60% | | | 82% | | | 69% | | |  |  |
| AD | 204 | | | 101 | | | 43 | | | 1168 | | | 91% | | | 83% | | | 67% | | | 74% | | |  |  |

**Table S3. Statistical tests for the age distribution**

| **Group A** | CN | MCI | AD |  | **Group B** | CN | MCI | AD |
| --- | --- | --- | --- | --- | --- | --- | --- | --- |
| Age_Mean | 72.5 | 72.1 | 78.1 |  | Age_Mean | 73.0 | 73.7 | 76.7 |
| Age_SD | 5.3 | 5.1 | 5.0 |  | Age_SD | 5.2 | 5.3 | 6.3 |
| p-value | CN-MCI | CN-AD | MCI-AD |  | p-value | CN-MCI | CN-AD | MCI-AD |
|  | 0.47 | 0.15 | 0.12 |  |  | 0.45 | 0.24 | 0.29 |
|  |  |  |  |  |  |  |  |  |
| **Group C** | CN | MCI | AD |  | **Group D** | CN | MCI | AD |
| Age_Mean | 74.0 | 75.3 | 76.9 |  | Age_Mean | 78.1 | 76.2 | 77.4 |
| Age_SD | 5.7 | 5.8 | 5.2 |  | Age_SD | 6.5 | 5.7 | 6.6 |
| p-value | CN-MCI | CN-AD | MCI-AD |  | p-value | CN-MCI | CN-AD | MCI-AD |
|  | 0.41 | 0.31 | 0.39 |  |  | 0.39 | 0.46 | 0.42 |

**Table S4. Results of the original analysis and those for female : male = 1 : 1**

| Self-recognition test (original) | | | | | | | | | | | | | | | | | | |
| --- | --- | --- | --- | --- | --- | --- | --- | --- | --- | --- | --- | --- | --- | --- | --- | --- | --- | --- |
|  | Group A | | |  | Group B | | |  | Group C | | |  | Group D | | |  | Correct | (%) |
| _Score_  _Exp._ | CN | MCI | AD |  | CN | MCI | AD |  | CN | MCI | AD |  | CN | MCI | AD |  |  |  |
| CN | 128 | 8 | - |  | 197 | 12 | 3 |  | 142 | 11 | 6 |  | 40 | - | - |  | 507 | 92 |
| MCI | 16 | 139 | - |  | 74 | 155 | 33 |  | 59 | 120 | 30 |  | 5 | 88 | 3 |  | 502 | 69 |
| AD | - | - | 13 |  | 6 | 1 | 60 |  | 7 | 2 | 75 |  | 1 | 3 | 79 |  | 227 | 91 |
|  |  |  |  |  |  |  |  |  |  |  |  |  |  |  |  |  |  |  |
| Self-recognition test (Female:Male=1:1) | | | | | | | | | | | | | | | | | | |
|  | Group A | | |  | Group B | | |  | Group C | | |  | Group D | | |  | Correct | (%) |
| _Score_  _Exp._ | CN | MCI | AD |  | CN | MCI | AD |  | CN | MCI | AD |  | CN | MCI | AD |  |  |  |
| CN | 101 | 7 | - |  | 143 | 9 | 6 |  | 133 | 9 | 6 |  | 18 | - | - |  | 432 | 91 |
| MCI | 19 | 125 | - |  | 59 | 142 | 27 |  | 26 | 97 | 19 |  | - | 68 | - |  | 582 | 74 |
| AD | - | - | 8 |  | 2 | 1 | 53 |  | 5 | 4 | 71 |  | - | - | 44 |  | 188 | 94 |
|  |  |  |  |  |  |  |  |  |  |  |  |  |  |  |  |  |  |  |
|  |  |  |  |  |  |  |  |  |  |  |  |  |  |  |  |  |  |  |
| Stratified 3-fold cross validation test (original) | | | | | | | | | | | | | | | | | | |
|  | Group A | | |  | Group B | | |  | Group C | | |  | Group D | | |  | Correct | (%) |
| _Score_  _Exp._ | CN | MCI | AD |  | CN | MCI | AD |  | CN | MCI | AD |  | CN | MCI | AD |  |  |  |
| CN | 122 | 14 | - |  | 177 | 28 | 7 |  | 133 | 20 | 6 |  | 31 | 6 | 3 |  | 463 | 84 |
| MCI | 33 | 122 | - |  | 97 | 130 | 35 |  | 68 | 103 | 38 |  | 9 | 75 | 12 |  | 430 | 59 |
| AD | 1 | - | 12 |  | 5 | 9 | 53 |  | 7 | 11 | 66 |  | 2 | 8 | 73 |  | 204 | 82 |
|  |  |  |  |  |  |  |  |  |  |  |  |  |  |  |  |  |  |  |
| Stratified 3-fold cross validation test (Female:Male=1:1) | | | | | | | | | | | | | | | | | | |
|  | Group A | | |  | Group B | | |  | Group C | | |  | Group D | | |  | Correct | (%) |
| _Score_  _Exp._ | CN | MCI | AD |  | CN | MCI | AD |  | CN | MCI | AD |  | CN | MCI | AD |  |  |  |
| CN | 91 | 17 | - |  | 118 | 35 | 5 |  | 123 | 18 | 7 |  | 16 | 2 | - |  | 432 | 81 |
| MCI | 28 | 116 | - |  | 80 | 118 | 30 |  | 43 | 74 | 25 |  | - | 66 | 2 |  | 582 | 64 |
| AD | - | - | 8 |  | 2 | 3 | 51 |  | 5 | 10 | 65 |  | - | 3 | 41 |  | 188 | 88 |
